# Factors Affecting Adverse Drug Reaction Reporting of Healthcare Professionals and Their Knowledge, Attitude, and Practice towards ADR Reporting in Nekemte Town, West Ethiopia

**DOI:** 10.1155/2016/5728462

**Published:** 2016-11-30

**Authors:** Lense Temesgen Gurmesa, Mohammed Gebre Dedefo

**Affiliations:** ^1^Nekemte Health Center, Oromia, Ethiopia; ^2^Department of Pharmacy, Wollega University, Oromia, Ethiopia

## Abstract

*Background*. Adverse drug reactions are global problems of major concern. Adverse drug reaction reporting helps the drug monitoring system to detect the unwanted effects of those drugs which are already in the market.* Aims*. To assess the knowledge, attitude, and practice of health care professionals working in Nekemte town towards adverse drug reaction reporting.* Methods and Materials*. A cross-sectional study design was conducted on a total of 133 health care professionals by interview to assess their knowledge, attitude, and practice using structured questionnaire.* Results*. Of the total respondents, only 64 (48.2%), 56 (42.1%), and 13 (9.8%) health care professionals have correctly answered the knowledge, attitude, and practice assessment questions, respectively. Lack of awareness and knowledge on what, when, and to whom to report adverse drug reactions and lack of commitments of health care professionals were identified as the major discouraging factors against adverse drug reaction reporting.* Conclusion*. This study has revealed that the knowledge, attitude, and practice of the health care professionals working in Nekemte town towards spontaneous adverse drug reaction reporting were low that we would like to recommend the concerned bodies to strive on the improvement of the knowledge, attitude, and practice status of health care professionals.

## 1. Introduction

Adverse drug reactions (ADRs) are global problems of major concern. They affect both children and adults with varying magnitudes, causing both morbidity and mortality [1–3]. An ADR is defined by the world health organization (WHO) as “a noxious, unintended effect of a drug that occurs in doses normally used in humans for the diagnosis, prophylaxis and treatment of disease” [4].

The information collected during the premarketing phase is incomplete with regard to adverse drug reactions and this is mainly because (1) patients used in clinical trials are limited in number and are not representative to the public at large. In addition, the conditions of use of medicines differ from those in clinical practice and the duration is limited. (2) Information about rare but serious adverse reactions, chronic toxicity, and use in special groups (such as children, the elderly, or pregnant women) or drug interactions is often incomplete. Therefore, postmarketing surveillance is important to permit detection of less common but sometimes very serious ADRs. Thus, postmarketing surveillance is important to permit detection of less common, but sometimes very serious ADRs. Therefore health professionals worldwide should report on ADRs as it can save lives of their patients and others [5].

Different studies have documented that new adverse reaction are discovered efficiently from spontaneous reporting than from other methods, including large postmarketing studies [3, 5–8]. The occurrences of ADRs depend on the age, sex, genetic, polypharmacy, dose accuracy, and environmental and other internal factors like disease conditions [9–20].

In Ethiopia, a report by Drug Administration and Control Authority (DACA), showed that out of the total of ADRs encountered (413) only 22 are reported to DACA making the total reported to be 5% only [12]. This shows that spontaneous reporting by health care professionals was very low. Other studies done in Ethiopia also suggested that awareness raising program on the ADR reporting system need to be designed to health professionals by relevant bodies and ADR reporting system need to be introduced to improve ADR reporting [9–11].

This study was aimed at investigating the knowledge, attitude, and practice of HCPs on spontaneous ADR reporting and factors affecting the reporting process in Nekemte town and also to suggest possible ways of improving method of reporting.

## 2. Methods

### 2.1. Study Setting and Period

Nekemte town is situated on a flat, hilly landscape. It is located at a distance of 331 km west of Addis Ababa, 110 km North East of Gimbi, the principal town of West Wollega Zone, and 250 km North West of Jima Zone in Oromia Regional state. According to the central statistical Agency of 2007, the population size of Nekemte is 75,219 [21]. The 2015 projected total population is estimated to be around 110,640 [22]. There are about 176 HCPs (clinical nurses, doctors, health officers, and pharmacists) working for health service in Nekemte town private and governmental health centers and clinics. The study was conducted from January 2015 to June 2015.

### 2.2. Study Design

Descriptive cross-sectional study design was conducted by using structure questionnaires.

### 2.3. Study Population

All the nurses, medical doctors, health officers, and pharmacists who are available during the study period and willing to participate in the study with equal chance were included.

### 2.4. Sample Size Determination

Sample size was calculated using single proportion of size less than 10,000 assuming the KAP of ADR-report to be 50%; to get maximum possible size the following equation was used:

(1) $$\begin{array}{c} S=\frac{z^{2}pq}{w}, \end{array}$$

where *S* is sample size, *z* ^2^ critical value equals 1.96, *p* is precision (marginal error) equal to 0.05, *S* = (1.96)^2^(0.5)/0.05^2^, and *S* = 384

The total number of HCPs (nurses, medical doctors, health officers, and pharmacists) working in Nekemte town private and governmental health centers and clinics as well as in Nekemte hospital is found to be 176.

Since this figure is below 10,000 we use the following adjustment for the sample size:

(2) $$\begin{array}{c} S=\frac{n}{1+n/N}, \end{array}$$

where *n* is sample size for population of size above 10,000 and *N* is number of source populations. Therefore,

(3) $$\begin{array}{c} S=\frac{384}{1+384/176}. \end{array}$$

*S* = 121; then by adding 10% to compensate nonrespondents, *S* = 133.

### 2.5. Sampling Technique

Assuming variation among the different HCPs KAP towards ADR reporting, study subjects were recruited using stratified random sampling technique with proportional allocation:

 medical doctors (MDs) = 25; then the sample taken was = 25/176*∗*133 = 19;
 nurses = 93; then the sample taken was = 93/176*∗*133 = 70;
 health officers (HOs) = 33; then the sample taken was = 33/176*∗*133 = 25;
 pharmacists = 25; then the sample taken was = 25/176*∗*133 = 19

### 2.6. Data Collection Process

Data were collected by the researcher assistants under the supervision of principal investigators using structural questionnaires on the sociodemographic status, the knowledge, attitude, and practice of health professional towards ADR reporting, and influencing factors.

### 2.7. Data Analysis and Interpretation

After data collection, data were entered into the Statistical Package for the Social Sciences (SPSS) version 20 for analysis. Checking, clearing, and coding of data were done before the analysis activities. Data collection from interviewee was analyzed, summarized, and represented in tables. By the analyzing data the KAP of HCPs towards ADR reporting and factors affecting the reporting process was assessed.

### 2.8. Ethical Consideration

Ethical clearance was obtained from the Ethical Review Committee of Wollega University, College of Medical and Health Sciences. A participant's written informed consent was obtained after explaining about the purpose and procedures of the study. In addition all the responses were kept confidential.

## 3. Results

This study was conducted on 133 health professionals comprising medical doctors, pharmacists, health officers, and nurses.

### 3.1. Sociodemographic Features

Of the total 133 HCPs, 90 (67.6%) were males and 43 (32.3%) were females. The majority of participants 98 (73.6%) were below 36 years. The majority of HCPs 50 (37.6%) have 3–5 years of service. The study included 70 (52.6%) nurses, 19 (14.3%) MDs, 19 (14.3%) pharmacists, and 25 (18.8%) HOs (Table 1).

### 3.2. KAP of Health Care Professionals

Regarding the knowledge of HCPs, 83 (62.4%) had heard about ADR reporting. Out of 83 HCPs who heard about ADR reporting, 37 (44.5%) get information about ADR reporting from formal teaching. Only 31 (37.4%) HCPs had heard about the existence of yellow card. Only 20 (24.0%) HCPs said that ADR had to be reported to Food, Medicine and Healthcare Administration and Control Authority of Ethiopia (FMHACA) (Table 2).

Regarding attitude of the HCPs, the majority of them (103 (77.4%)) agree that ADR reporting is essential and out of 103 HCPs 58 (43.6%) suggested that ADR reporting is encouraged when reaction is serious. Of the respondents 63 (47.5%) responded that premarket drug evaluation is not enough for detecting ADR. Most of the HCPs (77 (57.9%)) said that ADR reporting must be compulsory (Table 3).

Regarding practice of HCPs, only 36 (27%) HCPs had faced patients with ADR. From those 36 HCPs who have noticed ADRs from their clients, 14 (38.8%) have reported ADR; 11 (78.5%) had reported 1 to 3 times; and 3 (21.4%) did more than 3 times. On the reasons why HCPs did not report ADRs (i.e., from those 22), 10 (45.5%) were not aware whether to report them, 9 (40.9%) because there was no report available at the work places, and 2 (9%) do not know the system responsible for receiving their reports. Types of ADRs which were reported by 14 respondents are unexpected (5, 35.7%), serious (6, 42.8%), reactions to recently marketed (within five years) pharmaceuticals (2, 14.4%), and all of the three types (1, 7.1%). ADRs were reported mainly to the responsible bodies in the respective health center/hospital 7 (50.0%), to DTC 5 (35.7%), and FMHACA 2 (14.3%) (Table 4).

When we see the comparison of the KAP status of HCPs in the four professions, from the total of 20 KAP based questions interviewed, 9 (45%) were knowledge based, 5 (25%) attitudinal, and the remaining 6 (30%) practice oriented. Of the total respondents, only 64 (48.2%), 56 (42.1%), and 13 (9.8%) health care professionals have correctly answered the knowledge, attitude, and practice assessment questions, respectively (Table 5).

### 3.3. Factors That Affect Spontaneous ADR Reporting

#### 3.3.1. Discouraging Factor

As shown in Table 6, HCPs suggested the factors negatively affecting the ADR reporting process. Lack of awareness and knowledge on what, when, and to whom to report ADRs was 41 (30.8%) of the respondents followed by lack of commitments of HCPs constituting 34 (25.5%).

#### 3.3.2. Encouraging Factors

Factors responded by HCPs to improve ADR reporting as shown in Table 6 are awareness creation on what, when, and to whom to report ADRs accounting for 56 (42.1%) followed by in-service training 35 (26.3%).

## 4. Discussion

The result of our finding showed that the knowledge of HCPs about ADR reporting is low with only 64 (48.2%) of the HCPs having answered correctly to the knowledge based questions. This result is consistent with different studies; only 34.2% of the respondents had sufficient knowledge on the ADR reporting system in a study conducted in Amhara Region of Ethiopia [11], 23.17% in a study conducted at Southwest Ethiopia [9], 39.6% in a study conducted in Saudi Arabia [16], and 39.4% in a study conducted in Nepal [17].

When we compare the knowledge of HCPs among themselves medical doctors (84.2%) and pharmacists (84.2%) were more knowledgeable than health officers (56%) and nurses (25.7%). This finding is consistent with a study reported from Nepal [17]. However studies from Nigeria [13] and Saudi Arabia [16] reported that pharmacists are more knowledgeable than medical doctors and nurses. This shows that there is a knowledge variation between the different health care professionals on spontaneous ADR reporting which could be because of difference in access to information about ADR reporting.

Concerning the attitude of HCPs, only 56 (42.1%) HCPs have correctly answered the attitude based questions. The finding of this study showed that there is low attitude towards reporting ADR as compared to the previously done studies which showed high attitude towards ADR reporting: 75% in Southwest Ethiopia [9], 60% in Amhara Region of Ethiopia [11], 73.4% in different hospitals of Ethiopia [12], 66.3% in Nepal [17], and 82.2% in South India [20]. The difference could be because of lack of training, unawareness regarding the ADR reporting form, and lack of commitments of HCPs in West Wollega.

When we compare the attitude of HCPs among themselves pharmacists (89.5%) have good attitude towards ADR reporting followed by medical doctors (73.6%). Nurses have the poorest attitude in which only 20% have good attitude. This finding is consistent with a study reported from Southwest Ethiopia [9], Nigeria [13], and Nepal [17].

Regarding the practice of HCPs, this study revealed that the practice of HCPs towards ADR reporting is poor with only 13 (9.8%) of HCPs having answered correctly to the practice based questions. The practice of HCPs in this study is lower than other studies: 16.2% in Amhara Region of Ethiopia [11], 13.8% in different hospitals of Ethiopia [12], 33.7% in Nepal [17], and 22.8% in South India [20].

This study identified the factors that discourage the spontaneous ADR reporting of the HCPs; accordingly lack of awareness and knowledge on what, when, and to whom to report ADRs is the common factor followed by lack of commitments of HCPs and unavailable format. To overcome these discouraging factors HCPs have suggested some factors to improve ADR reporting; these are awareness creation on what, when, and to whom to report ADRs, in-service training, direct supervision of patients by pharmacist, and making report formats available are the main encouraging factors.

Strength of our study was that we have used a detailed structured questionnaire on KAP towards ADR reporting and the study subjects were recruited by using stratified random sampling technique with proportional allocation. The limitation of this study was that this study did not show the KAP difference of health care professionals within the same profession with different level of education.

## 5. Conclusion

The finding of this study showed that the knowledge, attitude, and practice of the HCPs working in Nekemte town towards spontaneous ADR reporting were low. Thus, we would like to recommend the concerned bodies to strive on the improvement of the knowledge, attitude, and practice status of health care professionals.

## Figures and Tables

**Table 1:** The sociodemographic status of respondents in Nekemte town from January 2015 to June 2015.

Variable	Category	Frequency	%
Age	<26	51	38.3
	26–35	47	35.3
	36–45	27	20.3
	>45	8	6.0

Sex	Male	90	67.6
	Female	43	32.3

Profession	MD	19	14.3
	Pharmacist	19	14.3
	Nurse	70	52.6
	Health officer	25	18.8

Year of service	<3	42	31.6
	3–5	50	37.6
	6–8	21	15.8
	>8	20	15.0

**Table 2:** The knowledge status data of HCPs on ADR reporting in Nekemte town from January 2015 to June 2015.

Variable	Category	Frequency	%
Heard about ADR-reporting	Yes	83	62.4
	No	50	37.6

Information source	In-service training	24	27.6
	Mass media	6	7.2
	Journals or publication	9	10.8
	Formal teaching	37	44.5
	Peer group	7	8.4

ADRs can be reported on	Drugs	43	51.8
	Medical devices	12	14.4
	Both	28	33.7

Know about existence of yellow card	Yes	31	37.4
	No	52	62.6

Agents to which ADR is to be reported	I do not know	28	33.7
	FMHACA	20	24.0
	Health center	14	16.8
	DTC/local drug monitor	9	10.7
	MD/physician	6	7.2
	Manufacturer	3	3.6
	Department of Pharmacy	3	3.6

Know responsible body	Yes	41	30.8
	No	92	69.1

To which drug do you expect more unexpected ADRs?	Newly marketed drugs	87	65.4
	Established drugs	14	10.5
	I do not know	32	24.0

ADRs that should be reported	All suspected reactions	20	15.0
	Unknown/unexpected	33	24.8
	Serious	40	30.1
	Unexpected therapeutic effects	21	15.7
	All	19	13.2

Mostly expected to be reported	Expected/labeled	45	33.8
	Unexpected/unlabeled	54	40.6
	I do not know	34	25.5

**Table 3:** Attitude of HCPs towards ADR-reporting in Nekemte town from January 2015 to June 2015.

Variable	Category	Frequency	%
ADR-reporting essential	Agree	103	77.4
	Disagree	6	4.5
	Neutral	24	18.1

Reporting encouraged when	Reaction is serious	58	43.6
	Unusual reaction	33	24.8
	You are certain	31	23.3
	Every one of each	11	8.3

Premarket drug evaluation enough	Yes	21	15.7
	No	63	47.5
	Neutral	49	36.8

Yellow card reporting cost effective	Yes	46	34.8
	No	13	9.8
	Neutral	74	55.6

ADR reporting	Compulsory	77	57.9
	Voluntary	38	28.6
	Neutral	18	13.5

**Table 4:** Practice of HCPs on ADR-reporting in Nekemte town from January 2015 to June 2015.

Variable	Category	Frequency	%
Faced ADR from patient/s/	Yes	36	27.0
	No	97	73.0

Reported	Yes	14	38.8
	No	22	61.1

Number of ADR-reports done	1–3 times	11	78.5
	More than 3 times	3	21.4

Reason not to report	I did not know	10	45.5
	No report form	9	40.9
	No system responsible	2	9.1
	Not usual to report	1	4.5

Types of ADRs-reported	Unexpected	5	35.7
	Serious	6	42.7
	Reaction to recently marketed	2	14.2
	All of the above	1	7.1

Agents to whom ADRs were reported	FMHACA	2	14.3
	DTC	5	35.7
	Other responsible bodies	7	50.0

**Table 5:** Comparison of the KAP of HCPs in each health profession in Nekemte town from January 2015 to June 2015.

Profession	Number of professionals	Correctly answered					
		Knowledge (9, 45% of total KAP questions)		Attitude (5, 25% of total KAP questions)		Practice (6, 30% of total KAP questions)	
		Frequency	%	Frequency	%	Frequency	%
Nurse	70	18	25.7	14	20.0	7	10.0
Doctor	19	16	84.2	14	73.6	2	10.5
Health officer	25	14	56.0	11	44.0	1	4.0
Pharmacist	19	16	84.2	17	89.5	3	15.8
Total	133	64	48.2	56	42.1	13	9.8
General assessment		Low knowledge		Low attitude		Low practice	

**Table 6:** Factors that affect spontaneous ADR-reporting in Nekemte town from January 2015 to June 2015.

Variables	Frequency	%
*Discouraging factors*		
Lack of awareness and knowledge on what, when, and to whom to report	41	30.8
Unavailable format	22	16.5
Not knowing or absence of responsible body	18	13.5
Lack of commitment of HCPs	34	25.5
Low patient follow-up/contact	12	9.0
I cannot suggest	6	4.5
*Encouraging factors*		
Awareness creation on what, when, how, & to whom to report and increasing awareness at all levels of education	56	42.1
In-service training	35	26.3
Make availability of the reports format	14	10.5
Announcing ADR report as it is a professional obligation of HCPs	8	6.0
Follow-up of patients	9	6.7
Direct supervision of patients by pharmacist	15	8.3
